# Case Report: An adult with *NCKAP1-*related neurodevelopmental disorder and autism spectrum disorder

**DOI:** 10.3389/fpsyt.2025.1532982

**Published:** 2025-06-09

**Authors:** Aruna Jain, Elizabeth VanSickle, Lia Zitano, Timothy Moss, Erica Schrader

**Affiliations:** ^1^ College of Human Medicine, Michigan State University College of Human Medicine, Grand Rapids, MI, United States; ^2^ Medical Genetics, Corewell Health Helen DeVos Children’s Hospital, Grand Rapids, MI, United States

**Keywords:** *NCKAP1*, neurodevelopmental disorder, autism spectrum disorder, nonsense variant, case report

## Abstract

**Introduction:**

We describe a 43-year-old man with neurodevelopmental disorder (NDD) with features of autism spectrum disorder (ASD) due to a rare pathogenic variant in the *NCKAP1* gene. There are only 5 young adults described in the literature with *NCKAP1*-related NDD; there are currently no reports of middle-aged or elderly adults with the condition. The most common clinical characteristics include ASD, intellectual disability (ID), speech-language problems, repetitive behaviors, and seizures.

**Conclusion:**

This case report highlights an adult phenotype of *NCKAP1*-related NDD with goals to 1.) contribute insight into a rare genetic variant leading to NDD with ASD features and 2.) highlight adult manifestations of *NCKAP1-*related NDD as a patient in middle adulthood with the condition has not yet been reported.

## Introduction

Neurodevelopmental disorders (NDD) are a group of complex conditions from atypical brain development that produce impairments or variations in behavior, cognition and communication (1). Autism spectrum disorder (ASD) is characterized by impaired social communication, restricted and repetitive behaviors, and sensory sensitivities. ASD affects individuals from early childhood and persists throughout their lives, and is an incredibly heterogeneous disorder with a wide range of severity and symptom presentation amongst patients. Thus, ASD is not a single clinical disorder, but rather a broad spectrum of phenotypes, with various genetic and environmental factors contributing to its etiology (2). The genetics of ASD are complex; there has thus far been over 1100 genes identified that are associated with ASD (3). Recent technological advances in genetics have solidified the notion that ASD is a product of a single gene variant, copy number variants, or chromosomal syndromes, rather than of Mendelian inheritance (4, 5). Due to the advent of exome sequencing, the contribution of single nucleotide variants has become clear as a large contributor to the etiology of ASD (6).

In this case report, we present an analysis of an adult with NDD associated with ASD who has a pathogenic single nucleotide variant in the *NCKAP1* gene. *NCKAP1* is a gene known to play a crucial role in neuronal development and migration as well as in neuronal cytoskeletal dynamics and neuronal differentiation (7). *NCKAP1* also is largely involved in formation of protein complexes essential for effective synaptic inhibition as well as actin dynamics (8). *NCKAP1* also interacts directly with *CYFIP2*, which has been linked to intellectual disability and ASD (9, 13). While the exact role of *NCKAP1* in the pathogenesis of NDDs and ASD is still not fully understood, research and documentation of *NCKAP1* variants as well as literature on intact *NCKAP1* supports that it is a gene that is likely to impact NDD and ASD risk. However, compared to other well-known genetic variants that lead to ASD, the literature on *NCKAP1* variants is limited, thus the neuromolecular findings on the role of intact *NCKAP1* is important to consider as it can help us to better understand why variants may lead to NDDs and ASD.

There are 36 reported individuals who have *NCKAP1* gene variants that explain their ASD (3). However, the majority of these cases are in children and adolescents; only 5 are adults, and they are from ages 20-23. Guo et al. (10) reported 21 of these 36 individuals, and they reported the core features of *NCKAP1*-related NDD to be ASD features, speech-language problems, childhood motor delay, intellectual disability (ID), and learning disabilities. Neuropsychiatric behaviors such as repetitive behavior, aggressive behavior, and attention deficit hyperactivity disorder (ADHD) were present in the majority of their participants as well. The summary and analysis of phenotypes by Guo et al. (10) has been useful in providing insight into the specific manifestations of an *NCKAP1* gene variant. This case report highlights an adult patient with a previous diagnosis of ASD, who was diagnosed with *NCKAP1*-related NDD in adulthood (due to recent inquiry of genetic testing). We aim to provide further insight into the possible clinical manifestations of *NCKAP1*-related NDD, specifically in an adult patient.

## Case presentation

The patient is a 43-year-old male who was adopted from first-degree consanguineous parents and was diagnosed with ASD at 17 years of age. Chart documentation indicates a diagnosis of Asperger’s syndrome at that time; however, the specific diagnostic assessments were not recorded. The delayed diagnosis may be attributed to barriers in accessing healthcare services during childhood. The patient also had been diagnosed with mild cognitive impairment, ID, ADHD, anxiety, and depression. These diagnoses were made through a community mental health agency external to our healthcare system, and detailed records regarding diagnostic criteria, assessment measures, severity, and longitudinal course were not available for review. An IQ score was likewise not recorded in the accessible medical record. Although the onset and severity of anxiety and depression could not be determined, medication history documents initiation of clonazepam and bupropion at 33 years of age. The only available quantitative measure of current symptom burden was a PHQ-4 administered at 41 years of age, which yielded a score of 4, suggestive of mild symptoms at that time.

At his medical genetics appointment, vital measurements were as follows: height 1.676 m (5’6”); weight 108 kg (238 lb); head circumference 55 cm (21.65”); BMI 38.41 kg/m². On physical examination, no craniofacial dysmorphic features were noted. His left upper extremity was larger than his right arm due to boggy subcutaneous tissue leading to increased girth. Neurological examination revealed a facial motor tic, no motor or focal deficits, and normal speech and comprehension.

The facial motor tic was first documented at 36 years of age. He is not receiving pharmacological treatment for the facial motor tic. The patient also had class 2 obesity, essential hypertension, obstructive sleep apnea, left upper extremity lymphedema, hyperlipidemia, and left ventricular hypertrophy (LVH). Obesity, hypertension, and obstructive sleep apnea were first noted at 33 years of age; hyperlipidemia and LVH at 34 years; and lymphedema at 36 years.

LVH was identified on both echocardiogram and cardiac magnetic resonance imaging (MRI).

The patient was diagnosed with non-congenital bilateral sensorineural hearing loss at 39 years of age following a hearing screen. He reported significant improvement with bilateral hearing aids. Audiometry was performed at 41 years of age, though no results were documented; however, his otolaryngologist described the hearing loss as bilateral sensorineural. Computed tomography (CT) of the internal auditory canals and posterior fossa at 40 years of age showed no structural abnormalities, supporting the diagnosis of sensorineural hearing loss. A summary of our patient's phenotypes is described in **Table 1**.

**Table 1:** Summary of our patient’s phenotypes in comparison to previous reports and unreported clinical manifestations of NCKAP1-Related Neurodevelopmental Disorder.

Phenotype seen in patient	Previously Reported?	Clinical Detail Notes
*Neurodevelopmental*		
ID	Yes	N/A
ASD	Yes	N/A
Facial motor tics	No	N/A
*Behavioral/Psychiatric*		
ADHD	Yes	N/A
Anxiety	Yes	N/A
Depression	Yes	N/A
Repetitive behavior	Yes	Patient reports habit of sucking on left first digit.
*Neurologic*		
Sensorineural hearing loss	Yes	Sensorineural was not specified, Guo et al. (10) used “hearing impairment.”
*Systemic*		
Obesity	Yes	N/A
Hypertension (HTN)	No	N/A
Obstructive sleep apnea	No	“Sleep disturbances” have been reported in Guo et al. (10), but not obstructive sleep apnea specifically.
Lymphedema of the left upper extremity	No	Lymphoscintography was abnormal in 2018, there was no lymphatic flow in the left upper extremity. Additionally, there was no known trauma or infection to the area, indicating congenital (primary) lymphedema.
Left ventricular hypertrophy (LVH)	No	1 case of congenital heart defect reported in Guo et al. (10), but is unspecified. Additionally, we are unaware of our patient’s onset of LVH (it was discovered in an echocardiogram at age 34). It is important to note HTN, which our patient has, is an etiology LVH
Gastrointestinal disturbances including hiatal hernia and esophagitis, secondary to chronic gastroesophageal reflux disease (GERD)	See notes	4 cases of gastrointestinal disturbance were reported in Guo et al. (10), but they were unspecified.
Hyperlipidemia	No	Patient takes Metoprolol which can be associated with hyperlipidemia.
Restless Leg Syndrome (RLS)	No	Patient takes Metoprolol which can be associated with RLS.

The patient initially desired genetic testing for a better understanding of his sensorineural hearing loss. Chromosome microarray was performed, and it showed no clinically significant copy number variants. However, the absence of heterozygosity was 18.69%, indicating first-degree relative consanguinity. The patient’s adoptive mother confirmed that his biological parents were siblings, and reported his biological father had a learning disability. Further details about the health status of biological parents is unknown. Due to the fact that the chromosome microarray did not indicate a cause for the patient’s hearing loss, ASD, or other medical conditions combined with the knowledge of his biological parents’ first-degree relationship, the patient and the medical genetics team opted to have exome sequencing performed.

Once the variant in *NCKAP1* was identified, the medical genetics team was prompted to further investigate manifestations of *NCKAP1* variants in order to best inform him on approaches to management and treatment. Because there is limited research on *NCKAP1* variants, interventions taken were focused on preventative care and included patient education and establishing care with specialists. The patient was counseled on the phenotypes reported in the literature, as well as the possibility of future children inheriting this genetic change. The risk of seizures stood out as a potentially urgent clinical feature for our patient to be aware of, and thus our team informed him of this potential risk and signs to monitor. Additionally, he is seeing a cardiologist routinely for his LVH. He also began seeing lifestyle medicine with goals to lose weight and modify his diet. Since the diagnosis, our patient is scheduled to follow up with medical genetics in two years, as no additional interventions beyond coordinating care with other specialties was necessary.

## Discussion

Individuals who have variants in *NCKAP1* may display variable expressivity, including NDD with ASD, ADHD, ID, neuropsychiatric behaviors, and seizures (10). Our patient’s clinical presentation aligns with some of the common phenotypes reported in literature on *NCKAP1* variants. Guo et al. (10) described two brothers, aged 21 and 22 years, with the same variant as our patient, c.2410C>T [p.Arg804*]. The variants were classified as likely pathogenic for the brothers, whereas the clinical significance of our patient’s variant is deemed pathogenic, likely due to the evidence from Guo et al. (10). The phenotypes that overlap between our patient and these brothers include ID and anxiety. Additionally, the proband was noted to be overweight, which aligns with our patient’s clinical profile. However, there are notable differences in the phenotypic expressions. The brothers exhibited features not present in our patient, including speech and language difficulties, aggressive behavior, and tall stature. The proband had seizures and skeletal abnormalities, while his brother had self-injurious behavior and microcephaly—none of which our patient demonstrated. Furthermore, ASD and ADHD, were either absent or unreported in the proband and his brother, respectively, highlighting the variability in expression even among individuals with the same genetic variant.

Many of the non-neurodevelopmental phenotypes observed in our patient have not yet been reported in the existing literature on *NCKAP1* variants. We describe them here to provide additional insights into the potential phenotypic spectrum of *NCKAP1* variants, particularly as individuals progress into adulthood. Of note, our patient’s presentation of localized lymphedema is significant, as primary lymphedema is a rare congenital condition most commonly associated with Turner syndrome. At 37 years of age, our patient underwent a lymphoscintigraphy study, which demonstrated absent lymphatic flow in the left upper extremity, consistent with congenital lymphedema. The patient reported no history of trauma, surgery, or infection involving the affected limb that might otherwise account for secondary lymphedema. He has experienced recurrent flares of lymphedema throughout his life, further supporting a diagnosis of primary lymphedema. Lymphedema associated with *NCKAP1* variants has not been previously reported. Based on the patient’s clinical presentation, absence of alternative etiologies, and lack of other pathogenic genetic variants, we propose localized lymphedema as a potential associated phenotype of *NCKAP1* variation.

Our patient’s diagnoses of hyperlipidemia, hiatal hernia, LVH, hypertension, and obstructive sleep apnea could potentially be secondary to obesity as well as advancing in age. Thus, we cannot say with certainty that he developed these diagnoses solely due to a *NCKAP1* variation. This highlights a limitation in the internal validity of our observations– namely, that multiple plausible etiologies exist for his findings, making it difficult to isolate the effect of the *NCKAP1* variant. However, obesity is supported as an association of *NCKAP1* variation in past reports, and thus, these conditions are essential to highlight, as screening for obesity-related comorbidities may be considered advisable. The external validity of our findings is limited by the fact that adult phenotypes associated with *NCKAP1* variation remain poorly characterized. Longitudinal follow-up studies, such as tracking the participants from Guo et al. (10) into adulthood, would strengthen the generalizability of observations like ours. More comprehensive data could help determine whether obesity and its related health outcomes are consistently part of the *NCKAP1*-related NDD spectrum or more coincidental in isolated cases.

It is also important to consider that our patient’s significant consanguinity, identified through microarray analysis and confirmed by his adoptive mother, may contribute to his phenotypic features through mechanisms that are not fully understood. This represents a potential confounding factor, as his clinical presentation may result from genetic influences independent of, or interacting with, the *NCKAP1* variant.

In addition to providing more insight into the adult clinical manifestations of *NCKAP1* variants, we wanted to note how this finding of the variant can be highlighted during genetic counseling of patients. Guo et al. (10) and Anazi et al. (11) show support for the variant arising *de novo* or inherited in the autosomal dominant pattern, so this would be a beneficial point to raise with patients as they consider having children of their own. Another point for genetic counselors to convey in the interest of informed decision-making and education is the variable expressivity of *NCKAP1-*related NDD seen in the literature, as limited information on the condition is available to the public.

Finally, this case highlights the fact that many adult patients with NDDs, even those diagnosed earlier in life, often have not undergone genetic testing. As a result, they may not have received condition-specific education, treatment, or preventative measures. As genetic testing becomes more accessible, the development of targeted intervention strategies for associated phenotypes, including preventative measures and specialty care not typically included in general health maintenance guidelines, becomes possible.

Exome sequencing, which identifies specific underlying gene variants responsible for NDDs, has proven beneficial for our patient’s treatment and for advancing the clinical understanding of the NDD spectrum (12). During discussions of the previously reported features of *NCKAP1*-related NDD, our patient shared that this aligns with his own experiences and feels that this explains the challenges he has faced. He understood the importance of following up with specialists and to seek care if new concerns, such as seizures, arise. He expressed enthusiasm and gratitude for having a biological explanation for his conditions and has a desire to connect with others affected by *NCKAP1*-related NDD.

## Methods

Exome sequencing was performed with GeneDx, and a single nucleotide variant was identified in the *NCKAP1* NM_205842 gene (c.2410 C>T in exon 23 p. [Arg804*]. This variant causes truncation in exon 24 in *NCKAP1.* This was interpreted by GeneDx as a pathogenic variant. The differential diagnosis supported by the GeneDx report as well as our patients phenotypes is *NCKAP1*-related neurodevelopmental disorder. Single nucleotide variants in *NCKAP1* have been reported in individuals with *NCKAP1*-related neurodevelopmental and neuropsychiatric disorders. Given this knowledge and the absence of other variants in exome sequencing, this patient’s *NCKAP1* variant was thought to be causative for his clinical symptoms and his diagnosis was felt to be consistent with a *NCKAP1*-related NDD. Our patient’s biological parents were not available for testing, so we were unable to determine if this patient’s *NCKAP1* variant was inherited or *de novo*.

## Conclusion

We describe the phenotype of an adult man who has *NKCAP1-*related NDD. By conducting exome sequencing and comparing his phenotype with the current literature, we were able to pinpoint the involvement of the *NCKAP1* gene in our patient’s NDD. The identification of *NCKAP1* as a risk gene for NDD highlights the need for continued research and reporting of its clinical presentation. Our patient’s case provides continued evidence for the association of *NCKAP1* gene variants leading to NDD associated with ASD, as well as unreported adult phenotypes. This finding not only has the potential to contribute to the growing body of knowledge surrounding the pathogenicity of *NCKAP1* variants, but it also helped to provide better self-understanding for our patient.

## Data Availability

The original contributions presented in the study are included in the article/supplementary material. Further inquiries can be directed to the corresponding author.
